# Reliability-based design and implementation of crow search algorithm for longitudinal dispersion coefficient estimation in rivers

**DOI:** 10.1007/s11356-021-12651-0

**Published:** 2021-03-08

**Authors:** Alireza Ghaemi, Tahmineh Zhian, Bahareh Pirzadeh, Seyedarman Hashemi Monfared, Amir Mosavi

**Affiliations:** 1 Department of Civil Engineering, University of Sistan and Baluchestan, Zahedan, Iran; 2 Water Security and Sustainable Development Hub, School of Engineering, Newcastle University, Newcastle upon Tyne, UK; 3 Faculty of Informatics, Selye Janos University, Komarom, 94501 Slovakia; 4 School of Economics and Business, Norwegian University of Life Sciences, 1430 Ås, Norway; 5 John von Neumann Faculty of Informatics, Obuda University, Budapest, 1034 Hungary

**Keywords:** Artificial intelligence, Crow search algorithm, Longitudinal dispersion coefficient, Machine learning, Monte Carlo simulation, Natural rivers, Reliability analysis

## Abstract

The longitudinal dispersion coefficient (LDC) of river pollutants is considered as one of the prominent water quality parameters. In this regard, numerous research studies have been conducted in recent years, and various equations have been extracted based on hydrodynamic and geometric elements. LDC’s estimated values obtained using different equations reveal a significant uncertainty due to this phenomenon’s complexity. In the present study, the crow search algorithm (CSA) is applied to increase the equation’s precision by employing evolutionary polynomial regression (EPR) to model an extensive amount of geometrical and hydraulic data. The results indicate that the CSA improves the performance of EPR in terms of *R*^2^ (0.8), Willmott’s index of agreement (0.93), Nash–Sutcliffe efficiency (0.77), and overall index (0.84). In addition, the reliability analysis of the proposed equation (i.e., CSA) reduced the failure probability (*P*_f_) when the value of the failure state containing 50 to 600 m^2^/s is increasing for the *P*_f_ determination using the Monte Carlo simulation. The best-fitted function for correct failure probability prediction was the power with *R*^2^ = 0.98 compared with linear and exponential functions.

## Introduction

Monitoring the contaminants of natural rivers is a fundamental part of environmental monitoring and assessment (Jeon et al. 2007; Rolsky et al. 2020). Developing novel methods for evaluating and accurately estimating water quality of rivers, as one of the fundamental freshwater recourses, has been an active research domain of environmental modeling and assessment (Parsaie and Haghiabi 2015). Urban and industrial sewages are globally known as the principal sources of rivers’ pollution (Sercu et al. 2009; Cheng 2003). Therefore, the study of mixing flow for reducing contamination level has drawn many researchers’ attention for water quality assessment (Hu et al. 2013; Haghiabi 2016). However, modeling the pollutant composition due to several uncertainties and irregularities regarding the formation of dead zones, recirculation mechanism, bed configuration, velocity, and secondary flow development is considered highly complex (Jeon et al. 2007). Modifying the longitudinal dispersion coefficient (LDC) has been used to specify the pollution density distribution (Li et al. 2020).

**Fig. 1:**
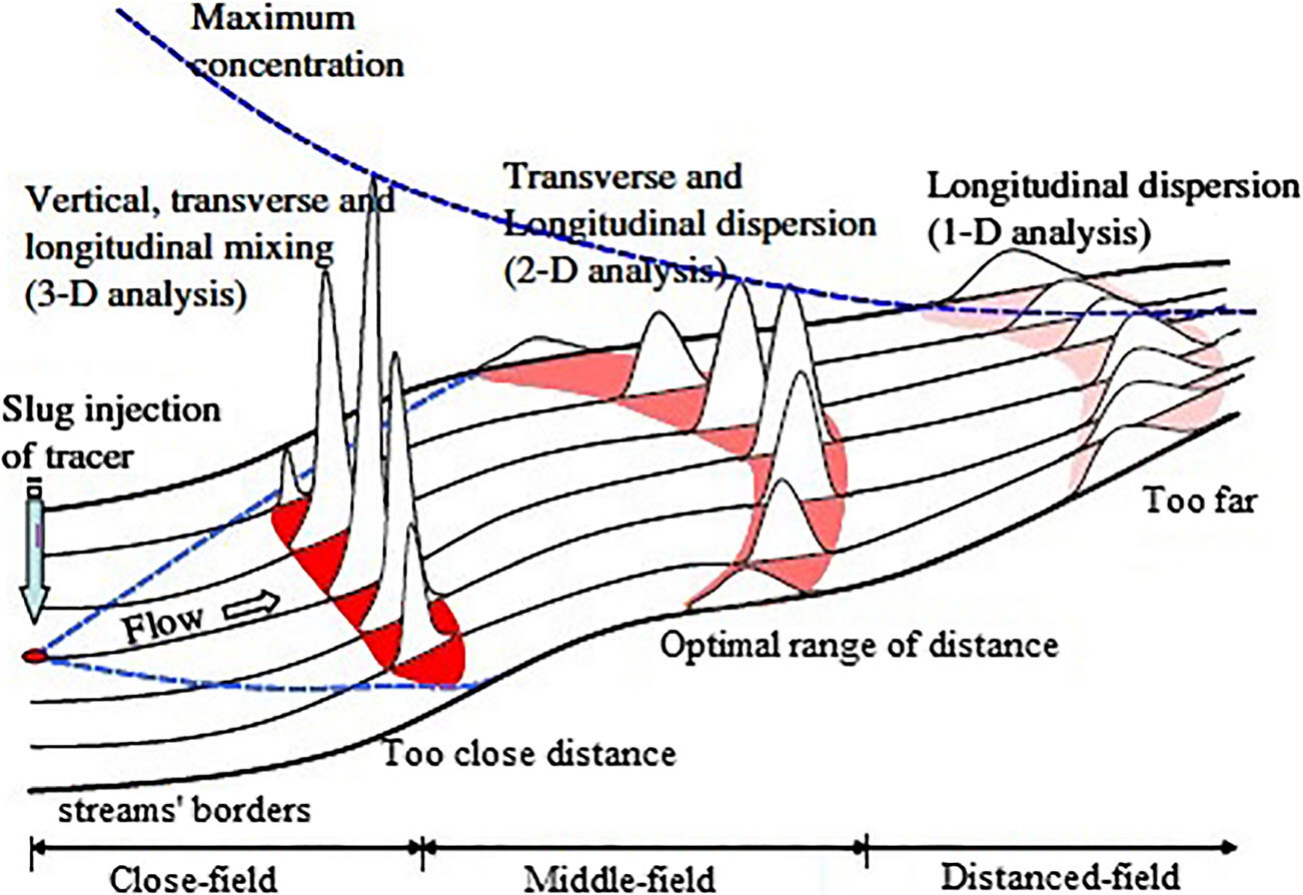
Conceptual diagram of dispersion mechanism in rivers

Various mixing stages influence a pollutant due to flow turbulence and molecular motion. As illustrated in Fig. 1, which is an illustration adapted from (Baek and Seo 2010), the pollutants gradually diffuse in the river and infect the downstream’s water. During the pollutant mixing process, firstly, vertical mixing rapidly occurs near the field (Seo and Cheong 1998). Afterward, a mixture occurs in the intermediate field in both longitudinal and transverse directions (Baek and Seo 2010). After completing the transverse mixing in natural rivers, the longitudinal mixing only is indefinitely maintained in the far field without any boundaries (Baek and Seo 2010). The dispersion coefficients are usually investigated using the concentration data collected from a tracer test. However, in the absence of any concentration dataset, the dispersion coefficients are determined by theoretical or empirical approaches based on the geometric and hydraulic parameters (Baek and Seo 2010). Empirical approaches and experimental datasets require time-consuming and expensive research; thus, there is an essential demand for professional tools for estimating this coefficient in rivers (Alizadeh et al. 2017c). Several studies (e.g., Elder 1959; Seo and Cheong 1998; Deng et al. 2001; Kashefipour and Falconer 2002; Disley et al. 2014; Zeng and Huai 2014; Sahin 2014; Wang and Huai 2016) estimate the LDC using the experimental methods and field measurements where the LDC of rivers represents the mixture’s intensity in the rivers (Alizadeh et al. 2017b). Among the parameters used for the prediction of LDC, hydraulic and geometrical river features, including channel width (*B*), flow depth (*H*), shear velocity (U_*_), and mean velocity (*U*), play prominent roles. However, the LDC estimations vary remarkably. Nevertheless, determining the environmental problems and evaluating the pollutant transport in rivers are important; consequently, it is important to estimate the LDC with a high accuracy (Alizadeh et al. 2017a). Generally, LDC measurement approaches have been divided into three categories: statistical equations, mathematical solutions, and artificial intelligence (AI) procedures. Mathematical solutions (e.g., numerical and analytical models) use the geomorphology and the channel’s geometry to estimate LDC. Statistical models apply the accessible measurement dataset for correlating the LDC based on the efficient parameters (regression analysis (RA) methods known as the most popular subcategories of statistical approaches). Because of the assumptions regarding the normality and linearity of this type of intricate phenomenon, these equations may not yield sufficiently accurate and valid results (Alizadeh et al. 2017c).

On the other hand, AI techniques have been recruited to overcome the disadvantage of regression-based methods in predicting various problems. In particular, machine learning methods, e.g., artificial neural networks (ANNs), support vector machine (SVM), model tree (MT), as well as metaheuristic algorithms, have recently shown promising results (Li et al. 2016; Wang et al. 2016; Zounemat-Kermani et al. 2016; Rezaie-Balf et al. 2017; Deo et al. 2018; Horton et al. 2018; Kisi et al. 2019; Najafzadeh and Ghaemi 2019; Fallah et al. 2019; Ghaemi et al. 2019; Maroufpoor et al. 2019; Bozorg-Haddad et al. 2019). In the case of LDC, although many studies (e.g., Adarsh 2010; Etemad-Shahidi and Taghipour 2012; Li et al. 2013; Najafzadeh and Tafarojnoruz 2016; Alizadeh et al. 2017b; Noori et al. 2017; Seifi and Riahi-Madvar 2019; Riahi-Madvar et al. 2019) have been performed during the last decades in order to predict this complicated phenomenon with high precision, the estimation results have not been adequately accurate or reliable. Consequently, this research aims to improve prediction performance by proposing the crow search algorithm (CSA) and evolutionary polynomial regression (EPR).

### Theoretical background

Despite describing the pollutant behavior in natural streams as a three-dimensional advection–diffusion equation (3D-ADE), which was obtained from a Fickian diffusion law, in the far downstream of the mixing zone where concentration variations in the horizontal and vertical directions have been insignificant, the 3D-ADE over width and depth yields

1 $$ \frac{\partial C}{\partial t}+U\frac{\partial C}{\partial x}=\mathrm{LDC}\frac{\partial^2C}{\partial {x}^2} $$

where *C* denotes the average cross-sectional concentration, *t* is time (seconds-based), and *U* and *x* are the average velocities of the cross-sectional and the longitudinal coordinate along the direction of mean flow, respectively (Noori et al. 2017; Rezaie-Balf et al. 2018).

Equation (1), which is taken into account as 1D advection–dispersion equation, has been highly recruited to evaluate the behavior of pollutants originating downstream from nonsteady point resources and is a balance between the advection and dispersion. The LDC is based on the river geometries, hydraulic condition, as well as fluid properties. The government parameters influencing the LDC are expressed as

2 $$ LDC=f\left(\rho, \mu, U,{U}_{\ast },H,W,{S}_{\mathrm{f}},{S}_{\mathrm{n}}\right) $$

where *ρ* and *μ* are fluid density and dynamic viscosity, respectively; the width of cross section and flow depth are shown by *W* and *H*, respectively; *S*_n_ is the sinuosity of the river, *S*_f_ is the longitudinal bed shape, and U_*_ denotes the shear velocity. To reach the dimensionless parameter in the LDC, the Buckingham theory was employed, and the dimensionless parameter was derived, as shown in Eq. (3) (Seo and Cheong 1998; Alizadeh et al. 2017a).

3 $$ \frac{LDC}{H{U}_{\ast }}=h\left(\rho \frac{UH}{\mu },\frac{U}{U_{\ast }},\frac{W}{H},{S}_{\mathrm{f}},{S}_{\mathrm{n}}\right) $$

Since the river flow is turbulent, the Reynolds number $\rho \frac{UH}{\mu }$ can be omitted and the measurement of the bed form and sinusitis path parameters cannot be clear. Consequently, their effectiveness can be selected as flow resistant, which can be observed in the flow depth. The nondimensional parameters that have been measured are

4 $$ \frac{LDC}{H{U}_{\ast }}=g\left(\frac{U}{U_{\ast }},\frac{W}{H}\right) $$

Developing a plethora of AI models and empirical formulas is mostly based on these nondimensionless parameters. Table 1 provides some well-known empirical formulas proposed by the researchers (Seo and Cheong 1998; Alizadeh et al. 2017c).

**Table 1:** Various techniques applied in LDC estimation

Author(s) (year)	Method	Equation	Evaluation metrics	Reference
Elder (1959)	Empirical	LDC = 5.93*U*_∗_*H*	–	Zeng and Huai (2014)
McQuivey and Keefer (1974)	Empirical	$\mathrm{LDC}=0.58 UB{\left(\frac{H}{U_{\ast }}\right)}^2$	–	Riahi-Madvar et al. (2009)
Fischer (1967)	Empirical	LDC = 0.011*HU*_∗_*AC*	–	Riahi-Madvar et al. (2019)
Liu (1977)	Empirical	LDC = 0.18*U*_∗_*H*(A)^2^(*C*)^0.5^	–	Sahay and Dutta (2009)
Koussis and Rodriguez-Mirasol (1988)	Empirical	LDC = 0.6*U*_∗_*HA*^2^	–	Riahi-Madvar et al. (2009)
Iwasa and Aya (1991)	Empirical	LDC = 2*U*_∗_*HA*^1.5^	–	Zeng and Huai (2014)
Seo and Cheong (1998)	Empirical	LDC = 5.915*U*_∗_*HA*^0.62^*C*^1.428^	*R*^2^	Deng et al. (2001)
Li et al. (1998)	Empirical	LDC = 0.2*A*^1.3^*C*^1.2^*HU*_∗_	–	Zeng and Huai (2014)
Deng et al. (2001)	Empirical	$$ {\displaystyle \begin{array}{l}\mathrm{LDC}=0.15{U}_{\ast }H\left(\frac{1}{8e}\right){A}^{1.667}{C}^2\\ {}e=0.145+\left(\frac{1}{3520}\right){A}^{1.38}C\end{array}} $$	*R*^2^	Deng et al. (2001)
Kashefipour and Falconer (2002)	Empirical	$\mathrm{LDC}=\left[7.428+1.775{A}^{0.62}{\frac{1}{C}}^{0.572}\right] CHU\mathrm{LDC}=10.612 UHC$	*R*^2^, ME, RMSE	Kashefipour and Falconer (2002)
Tayfur and Singh (2005)	ANN	–	*R*^2^, RMSE	Tayfur and Singh (2005)
Tayfur (2006)	Fuzzy, ANN, MLR	LDC = 0.906*Q* + 21.416	*R*^2^	Tayfur (2006)
Mohamed and Hashem (2006)	ANN	–	*R*^2^	Mohamed and Hashem (2006)
Toprak and Savci (2007)	Fuzzy logic	–	MSE, RMSE, SE, NE	Toprak and Savci (2007)
Toprak and Cigizoglu (2008)	ANN	–	*R*^2^, MSE, MRSE, SE, NE	Toprak and Cigizoglu (2008)
Riahi-Madvar et al. (2009)	ANFIS	–	*R*^2^, RMSE, MSE, MAE, ARE	Riahi-Madvar et al. (2019)
Sahay and Dutta (2009)	GA	LDC = 2*HU*_∗_*C*^1.25^*A*^0.96^	*R*^2^, RMSE, DR	Sahay and Dutta (2009)
Noori et al. (2009)	SVM-ANFIS	–	*R*^2^, RMSE, MAE	Noori et al. (2009)
Tayfur (2009)	GA	LDC = 0.91*Q* + 9.94	RMSE, MAE, DR	Tayfur (2009)
Adarsh (2010)	SVM and GP	–	*R*^2^, RMSE, MAE	Adarsh (2010)
Sahay (2011)	ANN	–	*R*^2^, RMSE, DR	Sahay (2011)
Noori et al. (2011)	ANN	–	*R*^2^, MAE	Noori et al. (2011)
Azamathulla and Wu (2011)	SVM	–	*R*^2^, RMSE	Azamathulla and Wu (2011)
Azamathulla and Ghani (2011)	GP	$$ \mathrm{LDC}={U}_{\ast }H\left(\begin{array}{l}\left({e}^{e^{\cos (c)+\left(\frac{(C)^2}{A+3.956}\right)}}\right)+\left(\frac{\sin (CA)\times (CA)}{e^{\sin A}}\right)\\ {}\left(\frac{C}{1.037}-\frac{A\times 10.76}{C-11.38}\right)\end{array}\right) $$	*R*^2^, RMSE	Azamathulla and Ghani (2011)
Etemad-Shahidi and Taghipour (2012)	M5 model tree	$$ \mathrm{LDC}=\left\{\begin{array}{l}15.49{HU}_{\ast }{A}^{0.78}{C}^{0.11}\kern2em \frac{B}{H}\le 30.6\\ {}14.12{HU}_{\ast }{A}^{0.61}{C}^{0.85}\kern2em \frac{B}{H}>30.6\end{array}\right. $$	RMSE, DR, ME	Etemad-Shahidi and Taghipour (2012)
Sahay (2013)	GP	LDC = 2*HU*_∗_*C*^1.37^*A*^0.72^*S*_*i*_^1.52^	*R*^2^, RMSE, DR	Sahay (2013)
Li et al. (2013)	DE	LDC = 2.2820*HU*_∗_*C*^1.4713^*A*^0.7613^	*R*^2^, RMSE, ARE	Li et al. (2013)
Tutmez and Yuceer (2013)	Kriging	–	*R*^2^, RMSE, MAE	Tutmez and Yuceer (2013)
Disley et al. (2014)	Empirical	$\mathrm{LDC}=3.563{HU}_{\ast }{\left(\frac{U}{\sqrt{gH}}\right)}^{-0.4117}{C}^{1.0132}{A}^{0.6776}$	*R*^2^, RMSE, NSE, WI	Disley et al. (2014)
Zeng and Huai (2014)	Empirical	LDC = 5.4*HUC*^0.13^*A*^0.7^	DR	Zeng and Huai (2014)
Sahin (2014)	Empirical	LDC = 48*C*^0.47^*R*_*h*_*U*	DR	Sahin (2014)
Toprak et al. (2014)	ANN	–	*R*^2^, MSE, SE	Toprak et al. (2014)
Sattar and Gharabaghi (2015)	GEP	$\mathrm{LDC}=8.45{HU}_{\ast }{A}^{0.5-0.514\mathrm{F}{\mathrm{r}}^{0.516}+C{0.42}^C}{C}^{1.65}$	*R*^2^, RMSE, NSE	Sattar and Gharabaghi (2015)
Parsaie and Haghiabi (2015)	ANN	–	*R*^2^, RMSE, MSE	Parsaie and Haghiabi (2015)
Antonopoulos et al. (2015)	Empirical and ANN	LDC = 0.000017625*C*^0.806657^*A*^3.93023^*HU*_∗_	ME, RMSE	Antonopoulos et al. (2015)
Haghiabi (2016)	MARS	–	*R*^2^, RMSE, MAE	Haghiabi (2016)
Najafzadeh and Tafarojnoruz (2016)	NF-GMDH-PSO	–	*R*^2^, RMSE, BIAS	Najafzadeh and Tafarojnoruz (2016)
Wang and Huai (2016)	Empirical	LDC = 17.648*HU*_∗_*C*^1.16^*A*^0.3619^	MER, MAE	Wang and Huai (2016)
Alizadeh et al. (2017c)	BN	–	*R*^2^, RMSE, DR, NSE	Alizadeh et al. (2017c)
Alizadeh et al. (2017b)	ANN (GA, ICA, BA, CSA, and LMA)	–	*R*^2^, RMSE, DR	Alizadeh et al. (2017b)
Alizadeh et al. (2017a)	PSO	$$ \mathrm{LDC}=\left\{\begin{array}{l}5.319{HU}_{\ast }{A}^{1.206}{C}^{0.075}\kern2em A\le 28\\ {}9.931{HU}_{\ast }{A}^{0.187}{C}^{1.802}\kern2em A\ge 28\end{array}\right. $$	NSE, RMSE, SE, DR	Alizadeh et al. (2017a)
Noori et al. (2017)	GC and NLR	LDC = *HU*_∗_*C*^1.305^*A*^0.856^*σ*^2.170^ (NLR)	*R*^2^, RMSE, MAE	Noori et al. (2017)
Rezaie-Balf et al. (2018)	EPR	$$ {\displaystyle \begin{array}{l}\mathrm{LDC}=+9.1941\frac{\ {U}^2\kern0.5em }{{BU_{\ast}}^2}\kern0.5em \exp\ \left(-1H+2U-2{U}_{\ast}\right)+\\ {}0.33128\frac{\kern0.5em {U}^{1.5}\ HB}{{U_{\ast}}^{0.5}}\kern0.5em \exp\ \left(-0.5{U}_{\ast}\right)+0\end{array}} $$	*R*^2^, RMSE	Rezaie-Balf et al. (2018)
Parsaie et al. (2018)	ANFIS-PCA	–	*R*^2^, RMSE	Parsaie et al. (2018)
Seifi and Riahi-Madvar et al. (2019)	ANFIS-GA ANN-GA	–	*R*^2^, RMSE, NSE, RAE, PI	Seifi and Riahi-Madvar et al. (2019)
Riahi-Madvar et al. (2019)	POMGGP	$$ \mathrm{LDC}={BU}_{\ast}\left(\begin{array}{l}0.01478+33.99{A}^{0.5}+8.497A{\left(\frac{1}{C}\right)}^2+\\ {}25.487\left(\frac{A}{C}\right)+\\ {}{A}^{1.5}{C}^4\left(0.0000486{A}^{0.5}-0.00021\right)\end{array}\right) $$	*R*^2^, RMSE, NSE, RAE, MAE	Riahi-Madvar et al. (2019)
Kargar et al. (2020)	SVR, M5P, RF, GPR	–	*R*^2^, RMSE, MAE	Kargar et al. (2020)
Memarzadeh et al. (2020)	SSMD-WOA	$$ \mathrm{LDC}=\left\{\begin{array}{l}0.2694{HU}_{\ast }{A}^{2.2456}\kern2em A\le 28\\ {}\left(0.35+\frac{8.7}{A}\right)\times \left(6.4+8A\right)\times {HU}_{\ast }{C}^{0.5}\kern2em A\ge 28\\ {}4.5{HU}_{\ast }{AC}^{0.5}\end{array}\right. $$	*R*^2^, RMSE, MAE, NSE	Memarzadeh et al. (2020)

### State of the art

This section employed various state-of-the-art scholarly studies on empirical and AI approaches for LDC prediction collected from the existing literature. A list of studies adopting empirical and AI techniques is presented in Table 1, which is arranged as an extensive overview of the prediction methods developed so far. It should be mentioned that in Table 1, the ratios of channel width to flow depth (*B*/*H*) and velocity to shear velocity (*U*/U_*_) are presented by *A* and *C*, respectively.

Elder (1959) proposed the first extension of Taylor’s approach for an open channel with infinite width using a laboratory dataset. He recruited a logarithmic velocity profile in the vertical direction and introduced an equation (Alizadeh et al. 2017b). Fischer (1967) suggested a simplified integral equation which presented the advantage of LDC estimation in the nondimensional form of accessible parameters. Liu (1977) considered lateral velocity gradients’ role in the LDC and suggested an expression in natural streams.

Seo and Cheong (1998) suggested an empirical equation with respect to the one-step technique developed by Huber (1981); it was a sturdy regression approach with a permissible estimating even at the presence of moderately bad leverage points. They used 59 sets of data from 26 U.S. streams to implement their equation. Their findings revealed that their equation had outperformed in comparison with other existing expressions. Deng et al. (2001) derived expressions for LDC prediction by assuming the importance of the transverse turbulent mixing (e). Based on the dimensional and regression analysis, Kashefipour and Falconer (2002) developed a predictive equation to estimate the LDC in natural rivers using 81 datasets collected from 30 rivers in the USA.

Disley et al. (2014) presented a predictive equation for a LDC using combined datasets from 29 rivers. Based on the outcomes, they concluded that their proposed equation was far superior to other empirical equations. Additionally, they found that the Froude number played a key role in capturing the effect of slope of the reach. Furthermore, Zeng and Huai (2014) established an empirical formula to estimate the LDC based on the 116 datasets of width, depth, cross-sectional averaged velocity, and bed shear velocity. Based on the results, their formula was as an effective method for LDC prediction. The evaluation performed by a couple of empirical approaches concerning 128 field datasets collected from 41 natural rivers in the USA revealed that the empirical equation obtained by Sahin (2014) was more valid and reliable than other predictive methods for LDC estimation in rivers.

In a research by Hamidifar et al. (2015), the examination of longitudinal dispersion in a compound open channel was performed for both vegetated and smooth floodplains and various flow conditions. They concluded that the magnitude of LDC had an increasing trend by implanting vegetation over the floodplain as well as increasing the relative flow depth. Outcomes of two studies by Farzadkhoo et al. 2018, 2019a) indicated that roughening the floodplain with stems was one of the important factors in increasing the longitudinal flow velocity and the Reynolds shear stress in the main channel. The maximum value of nondimension (LDC/*U*_⁎_*H*) was also found at the bend apex. Moreover, by increasing the relative flow depth, the nondimensional LDC (LDC/*U*_⁎_*H*) values decreased in the compound meandering channel for all the vegetated cases.

Furthermore, Farzadkhoo et al. (2019b) investigated the effect of rigid vegetation on the LDC estimation in a compound open channel. According to the results, floodplain vegetation caused the depth-averaged longitudinal velocity and LDC values to decrease and increase, respectively, compared with nonvegetated conditions. The results of a study by Shin et al. (2020) indicated that the cross-sectional averaged values of the dimensionless LDC, determined by the velocity profile data in a range of 4.1–6.5, had a behavior corresponding to the theoretical values, whereas this value, by a concentration data between 14.7 and 35.5, was 4–6 times greater than the velocity-based coefficient.

In terms of artificial intelligence, Tayfur and Singh (2005) used AI methods in LDC prediction for the first time. They employed an artificial neural network to model the LDC by 71 data of geometric and hydraulic parameters. The results showed that ANN could predict this target better than the empirical methods. Moreover, fuzzy, ANN, as well as MLR were applied by Tayfur (2006) to estimate the LDC based on 92 datasets of field dataset. He demonstrated that the fuzzy approach had higher performance compared to the other predictive methods.

Adarsh (2010) evaluated the degree of precision of data-driven models, including SVM and genetic programming (GP) in LDC estimation. The results indicated the superiority of the GP model to SVM and empirical methods. MT was employed by Etemad-Shahidi and Taghipour (2012) to estimate the LDC. In their study, for developing the proposed model, 149 distinctive hydraulic and geometrical field datasets of several rivers were applied. The error criteria confirmed that MT demonstrated a significantly good performance in capturing the relationship between input and output variables for LDC prediction than empirical approaches. The accuracy of the GP expression implemented by Sahay (2013) indicated that the GP model outperformed the empirical methods (e.g., Fisher and Liu) to predict LDC. They also found that the channel sinuosity was considered as a critical input variable for LDC prediction.

Sattar and Gharabaghi (2015) used 150 geometric and hydraulic datasets at hand for LDC prediction. Their study illustrated that the gene expression programming (GEP) model yielded the best performance. Najafzadeh and Tafarojnoruz (2016) evaluated the performance of the neuro-fuzzy–based group method of data handling (NF-GMDH)–particle swarm optimization (PSO) compared to some of the approaches such as MT, genetic algorithm (GA), and DE in LDC prediction. In their study, NF-GMDH had better accuracy than other alternative methods. They performed sensitivity analysis (SA) to select the important variables in LDC prediction. They also concluded that the flow depth had the most effective performance on the target variable. In a study by Alizadeh et al. (2017a), a multi-objective PSO algorithm was applied to derive a new expression in order to prognosticate the LDC. Based on the results, PSO methodology could increase the precision of the predictive equations by considering the optimum coefficient values.

Rezaie-Balf et al. (2018) developed evolutionary polynomial regression to estimate the LDC. According to statistical measures, EPR was an appropriate tool in comparison to the other alternative methods (e.g., PSO, GA, and MT). In addition, the result of sensitivity analysis demonstrated that channel width played a prominent role in LDC estimation. Evaluation of support vector regression (SVR), M5P, Gaussian process regression (GPR), and random forest (RF) was performed by Kargar et al. (2020) to estimate the LDC in the natural streams. Their findings illustrated that the applied M5P model outperformed the other alternative methods. Whale optimization algorithm (WOA) was applied by Memarzadeh et al. (2020) to improve the accuracy of the LDC predictive equation. Their outcomes illustrated that the proposed method could be considered as a useful method to estimate LDC.

**Fig. 2:**
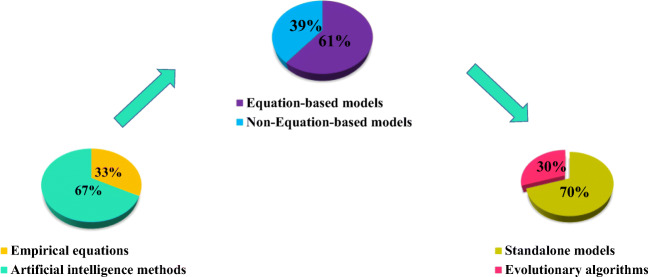
Different techniques applied in LDC estimation

In general, in recent years, the LDC prediction has been performed using AI (67%) and empirical (33%) methods (Fig. 2). In terms of AI techniques, approximately 39% of the utilized methods have the formula to predict the LDC. Additionally, only few studies in LDC prediction (30% of equation-based models) have been carried out on the basis of the evolutionary algorithms.

### Objective

Since the LDC is considered as a complicated phenomenon, obtaining a predictive model with an acceptable level of accuracy has attracted many researchers’ attention. As a result, a number of predictive approaches based on the empirical and AI methods have been reviewed to find the best approach. As the first attempt, this study aims to provide a comprehensive overview of applied LDC estimation techniques. Secondly, the main contribution of the present research is to improve one of the LDC equations (Rezaie-Balf et al. 2018) by using a kind of metaheuristic algorithm called CSA. It can be said that there is no published study related to employing this algorithm in LDC prediction. The accuracy of the proposed model is evaluated with other existing equations that are provided for LDC predictions. Thirdly, after selecting the best-fitted model in LDC estimation using conventional metrics, the partial derivative sensitivity analysis (PDSA) is applied for evaluating the pattern of input variables by the superior model. Afterward, the failure limit of a phenomenon is defined as a permissible domain for its safety. Different items (e.g., number of input variables) may influence the appropriate failure limits. One of the reliability evaluation techniques is Monte Carlo simulation (MCS) which is recruited in this study to determine the failure probability of the best LDC predictive equation in different failure states. Eventually, the variations of failure probabilities regarding the average and standard deviation corresponding to the suitable distribution of input variables are investigated.

## Proposed models

### Crow search algorithm

Among different types of animals and birds, crows are the most intelligent, and despite the small size of their brains, they have longer memories. They can communicate in sophisticated ways, memorize faces using tools, hide foods, and remember their positions during the various seasons. These features cause crows to discover and steal other crows’ hidden foods when they are not there. If a crow finds out that it is being followed by another one, it attempts to misguide the follower by flying to another area. Considering this, Askarzadeh (2016) introduced the CSA as a novel evolutionary algorithm to solve sophisticated optimization difficulties. This approach follows four principles as follows:

1. Their lives are as herd form
2. They maintain the location of the hidden food
3. They pursue each other for robbery
4. Crows memorize their caches from being pilfered using a probability

Like other algorithms, the optimization process begins with a dimensional environment containing the crow number (or population size). Suppose *x* denotes the position of crow *i* at each time (iteration) in the search area, which is calculated using a vector ${x}^{i,\mathrm{iter}}=\left[{x}_1^{i,\mathrm{iter}},{x}_2^{i,\mathrm{iter}},\dots, {x}_d^{i,\mathrm{iter}}\right]$ where *i* = (1, 2, …, *N*) and *i* = (1, 2, …, *N*). Each crow keeps in mind the position of its hidden location. It can be said that the best place of the hidden food experienced by each crow is preserved in its memory. Therefore, the crow’s hiding position *i* in iteration iter is the crow memory, which is illustrated by *m*^*i*, iter^. In each iteration, two states can occur, crow *j* flies to its hiding position (*m*^*j*, iter^), and crow *i* follows crow *j* to discover the hidden place of crow (Askarzadeh 2016; Díaz et al. 2018):

1. If crow *j* does not recognize that it is followed by crow *i*, crow *i* finds out the hidden place of crow *j*. Hence, the new position of crow *i* is expressed as

5 $$ {x}^{i,\mathrm{iter}+1}={x}^{i,\mathrm{iter}}+{r}_i\times \mathrm{f}{\mathrm{l}}^{i,\mathrm{iter}}\times \left({m}^{j,\mathrm{iter}}-{x}^{i,\mathrm{iter}}\right) $$

where fl^*i*, iter^ is the flight length for crow *i* in iteration iter and *r*_*i*_ presents a random number of the uniform distribution in an interval of 0 and 1. If fl value is considered less than 1, it brings about a local search and provides other situation of crow *i* between *x*^*i*, iter^ and *m*^*j*, iter^; otherwise, a global search will be anticipated, which causes the next situation of crow *i* gained away from *x*^*i*, iter^ and may exceed *m*^*j*, iter^.

(2) If crow *j* becomes aware that crow *i* is pursuing it to get its hidden food, it will deceive crow *i* by changing the food situation. States 1 and 2 are written briefly as

6 $$ {x}^{i,\mathrm{iter}+1}=\left\{\begin{array}{c}{x}^{i,\mathrm{iter}+1}={x}^{i,\mathrm{iter}}+{r}_i\times \mathrm{f}{\mathrm{l}}^{i,\mathrm{iter}}\times \left({m}^{j,\mathrm{iter}}-{x}^{i,\mathrm{iter}}\right){r}_j\ge \mathrm{A}{\mathrm{P}}^{i,\mathrm{iter}}\\ {}\mathrm{a}\ \mathrm{random}\ \mathrm{position}\ \mathrm{otherwise}\ \end{array}\right. $$

where AP^*i*, iter^ indicates the awareness possibility of crow *j* at iteration iter. The function of this parameter is balancing the exacerbation and variety for increasing the exacerbation by minor quantity for awareness probability by searching a local space and rising the probability value of the awareness, and CSA tends to investigate the searching space on the global scale. In sum, crow search algorithm implementation in solving the optimization problems can be expressed as (Askarzadeh 2016; Rezaie-Balf et al. 2019):

1. Defining the optimization problem and its constraints, selecting the CSA flock size (*N*), decision variables, the awareness probability (AP), the number of iteration (iter_max_), as well as the length of flight (fl).
2. Randomly finding the memory and position in a *d*-dimensional search space for proposed crows based on Eqs. (7) and (8). Each crow is considered as a conceivable solution for a specific problem, and *d* reveals the values for decision variables.

7 $$ \mathrm{Position}=\left[\begin{array}{c}\begin{array}{ccc}{x}_1^1& {x}_2^1& \dots \\ {}{x}_1^2& {x}_2^2& \dots \\ {}\vdots & \vdots & \vdots \end{array}\ \begin{array}{c}{x}_d^1\\ {}{x}_d^2\\ {}\vdots \end{array}\\ {}{x}_1^N\kern0.5em {x}_2^N\dots \kern0.5em {x}_d^N\end{array}\right] $$

8 $$ \mathrm{Memory}=\left[\begin{array}{c}\begin{array}{ccc}{m}_1^1& {m}_2^1& \dots \\ {}{m}_1^2& {m}_2^2& \dots \\ {}\vdots & \vdots & \vdots \end{array}\ \begin{array}{c}{m}_d^1\\ {}{m}_d^2\\ {}\vdots \end{array}\\ {}{m}_1^N\kern0.5em {m}_2^N\ \begin{array}{cc}\dots & {m}_d^N\end{array}\end{array}\right] $$

3. Fitness function evaluation of each crow using the decision variables putting into the objective function.
4. Generating a new position by crow *i* which one crow (crow *j*) selects randomly and chasing it for finding crow *j*’s hidden food resource (Eq. (5)).
5. Evaluating the possibility of the new situation by all crows. If the possibility of a new position of each crow is confirmed, updating the position of that crow is conducted. Otherwise, the crow stays in that situation and does not generate a new position.
6. Afterwards, the fitness function is assessed for the new position of each crow.
7. Eventually, the memory of crows is updated using Eq. (9)

9 $$ {m}^{i,\mathrm{iter}+1}=\left\{\begin{array}{c}{x}^{i,\mathrm{iter}+1}\ f\left({x}^{i,\mathrm{iter}+1}\right)\ \mathrm{is}\ \mathrm{better}\ \mathrm{than}\ f\left({m}^{i,\mathrm{iter}}\right)\\ {}{m}^{i,\mathrm{iter}}\kern1.5em \mathrm{otherwise}\ \end{array}\right. $$

where the objective function is represented by *f* (.), and *x*^*i*, iter^ and *m*^*i*, iter^ are the position and memory of crow *i* in iteration iter, respectively. The termination benchmark is evaluated (repeating steps 4–7 until iter_max_). Ultimately, the optimum solution is the best memory position calculated based on the objective function (Askarzadeh 2016; Rezaie-Balf et al. 2019).

### Geometry and hydraulic parameters influencing LDC

In this research, in order to implement CSA for estimating the LDC, a comprehensive field dataset including flow velocity, flow depth, channel width, and shear velocity was collected from the previous literature (e.g., Etemad-Shahidi and Taghipour 2012). This dataset has been applied to predict LDC in a wide range of the former studies with access to a huge number of natural streams. Moreover, it is obvious that these parameters have remarkably influence the LDC estimation (Noori et al. 2016). In general, 149 distinctive data records containing various hydraulic and geometric parameters have been applied in the model implementation (Etemad-Shahidi and Taghipour 2012). Moreover, in this study, among the defined distributions in MATLAB software, the proper distribution of each input variable based on the Kolmogorov–Smirnov test has been calculated. Hence, results of the statistical analysis of the data used (average (mean), maximum (max), minimum (min), standard deviation (SD), and their suitable distributions) are shown in Table 2.

**Table 2:** Statistical indices of the parameters applied for the EPR-CSA technique

Parameters	*B* (m)	*H* (m)	*U* (m/s)	U_*_ (m/s)	LDC (m^2^/s)
Maximum	253.6	8.2	1.73	0.55	1486.5
Minimum	1.4	0.14	0.029	0.0016	0.2
Average	49.58	1.35	0.47	0.08	83.29
SD	48.44	1.32	0.31	0.07	181.56
Distribution	Lognormal	Generalized extreme value	Generalized extreme value	Generalized extreme value	Log-logistic

It is clear that the largest maximum value of LDC (1486.5 m^2^/s) is roughly twice the second largest value. Therefore, removing these values is a convenient way (Tayfur and Singh 2005; Li et al. 2013; Disley et al. 2014). Despite the fact that omitting the largest LDC value may improve the model precision, it limits the implemented model usage. Accordingly, this study aims to improve the existing LDC equation provided by the EPR model using CSA.

### Development of CSA in the prediction of the LDC

As for the artificial intelligence methods (e.g., ANN, GEP, and MT), parameter selection is one of the prominent processes for getting better performance of the methods. For illustration, in terms of ANN, the weight and the number of hidden layers are used to optimize this model. Even an incorrect selection of optimized parameters leads to worse performance of the model in comparison to what expected. As a result, applying the metaheuristic approaches can be a worthwhile way for one has no longer count on a deep experience on the application of each method to the problem.

On the other hand, as mentioned above, in the recent decades, the LDC prediction has drawn the attention of lots of researchers. Thus, various methods have been recruited to estimate the LDC accurately. Among the applied approaches, most of which are presented in Table 1, EPR is considered as one of the successful tools in LDC prediction. In this regard, the principal aim of this study is to exhibit the usage of CSA to optimize the LDC equation gained from EPR.

EPR, as one of the artificial intelligence techniques, is the nonlinear global stepwise regression, which presents mathematical expressions according to the evolutionary calculation. EPR also applied GA along with numerical regression for improving the mathematical equations to calculate optimum parameters. The common form of EPR mathematical equations is written as (Giustolisi and Savic 2009; Kakoudakis et al. 2017)

10 $$ y={\sum}_{i=1}^m\kern1em F\left(X,f(X),{a}_i\right)+{a}_0 $$

where *y* indicates the estimated value; *a*_*i*_ and *X* are the constant coefficient and input variables, respectively; *m* is the sample number; *F* creates model structures in the process; and *f* is the user-defined function.

Finally, EPR expression can be presented based on one of the general forms as below

11 $$ y={a}_0+{\sum}_{j=1}^m\kern1em {a}_j{\left({X}_1\right)}^{\mathrm{ES}\left(j,1\right)}\dots {\left({X}_K\right)}^{\mathrm{ES}\left(j,K\right)}f\left({\left({X}_1\right)}^{\mathrm{ES}\left(j,K+1\right)}\right)\dots f\left({\left({X}_K\right)}^{\mathrm{ES}\left(j,2K\right)}\right) $$

12 $$ y={a}_0+{\sum}_{j=1}^m\kern1em {a}_jf\left({\left({X}_1\right)}^{\mathrm{ES}\left(j,1\right)}\dots {\left({X}_K\right)}^{\mathrm{ES}\left(j,K\right)}\right) $$

13 $$ y={a}_0+{\sum}_{j=1}^m\kern1em {a}_j{\left({X}_1\right)}^{\mathrm{ES}\left(j,1\right)}\dots {\left({X}_K\right)}^{\mathrm{ES}\left(j,K\right)}f\left({\left({X}_1\right)}^{\mathrm{ES}\left(j,K+1\right)}\right)\dots {\left({X}_K\right)}^{\mathrm{ES}\left(j,2K\right)} $$

14 $$ y=f\left({a}_0+{\sum}_{j=1}^m\kern1em {a}_j{\left({X}_1\right)}^{\mathrm{ES}\left(j,1\right)}\dots {\left({X}_K\right)}^{\mathrm{ES}\left(j,K\right)}\right) $$

where ES(*j*, *K*) indicates a function exponent which is related to the *K*th input of the *j*th term, and its bound is assigned by user (Khosravi and Javan 2019; Balacco and Laucelli 2019).

By assuming dimensional analysis in LDC estimation on the basis of the hydraulic (including velocity (*U*) and shear velocity (*U*_∗_)) and geometric (including channel width (*B*) and flow depth (*H*)) parameters, Eq. (15) is obtained

15 $$ LDC=f\left(B,H,U,{U}_{\ast}\right) $$

Additionally, the EPR mathematical equation that is provided for LDC estimation is written as

16 $$ LDC=+9.1941\frac{U^2\kern2em }{B{U_{\ast}}^2}\kern.2em \exp \kern.2em \left(-1H+2U-2{U}_{\ast}\right)+0.33128\frac{\kern.2em {U}^{1.5}\kern.2em HB}{{U_{\ast}}^{0.5}}\kern.2em \exp \kern.2em \left(-0.5{U}_{\ast}\right)+0 $$

**Fig. 3:**
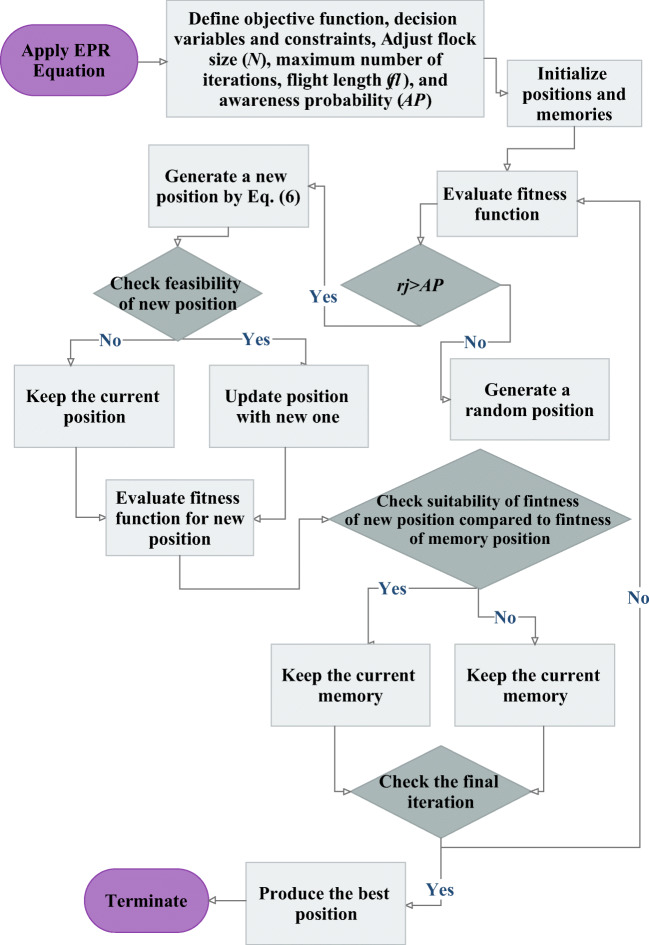
LDC estimation diagram using the EPR-CSA model

By considering Eq. (16), the general expression of the LDC is written as follows:

17 $$ LDC=+a\frac{U^b\kern.2em }{B^c{U_{\ast}}^d}\kern.2em \exp \kern.2em \left(- eH+ fU-g{U}_{\ast}\right)+h\frac{\kern.2em {U}^i\kern.2em {H}^j{B}^k}{{U_{\ast}}^l}\kern.2em \exp \kern.2em \left(-m{U}_{\ast}\right)+n $$

where *a*, *b*, and *c* until *n* are constant values of the equation. Therefore, the major purpose of this research is to use the CSA to gain the constant optimum values. The adjustable CSA parameters are shown in Table 3. These parameters included the flock size (*N*), maximum number of iterations (iter_max_), flight length (fl), and AP, which are determined using trial and error methodology and demonstrated optimum values of this study. In addition, LDC estimation diagram using EPR-CSA model is illustrated in Fig. 3.

**Table 3:** Characteristics of the developed CSA

Flock size	Maximum number of iterations	Flight length	Awareness probability
149	1000	0.5	0.3

## Model assessment criteria

In the current study, the performance of the predictive methods is assessed by a couple of conventional benchmarks consisting of determination coefficient (*R*^2^), root mean square error (RMSE), Willmott’s index of agreement (WI), mean absolute error (MAE), Nash–Sutcliffe efficiency (NSE), overall index (OI), and objective function (OBJ), which are written as

18 $$ {R}^2=\left(\frac{\sum_{I=1}^N\left({\mathrm{LDC}}_{\mathrm{Pre}}^i-{\mathrm{LDC}}_{\mathrm{Pre}}^{\mathrm{mean}}\right)\left({\mathrm{LDC}}_{\mathrm{Obs}}^i-{\mathrm{LDC}}_{\mathrm{Obs}}^{\mathrm{mean}}\right)}{\sqrt{\sum_{i=1}^N{\left({\mathrm{LDC}}_{\mathrm{Obs}}^i-{\mathrm{LDC}}_{\mathrm{Obs}}^{\mathrm{mean}}\right)}^2{\sum}_{i=1}^N{\left({\mathrm{LDC}}_{\mathrm{Pre}}^i-{\mathrm{LDC}}_{\mathrm{Pre}}^{\mathrm{mean}}\right)}^2}}\right) $$

19 $$ \mathrm{RMSE}=\sqrt{\frac{\sum_{i=1}^N{\left({\mathrm{LDC}}_{\mathrm{Obs}}^i-{\mathrm{LDC}}_{\mathrm{Pre}}^i\right)}^2}{N}} $$

20 $$ \mathrm{NSE}=1-\frac{\sum_{i=1}^N{\left({\mathrm{LDC}}_{\mathrm{Obs}}^i-{\mathrm{LDC}}_{\mathrm{Pre}}^i\right)}^2}{\sum_{i=1}^N{\left({\mathrm{LDC}}_{\mathrm{Obs}}^i-{\mathrm{LDC}}_{\mathrm{Obs}}^{\mathrm{mean}}\right)}^2} $$

21 $$ \mathrm{WI}=1-\frac{\sum_{i=1}^N{\left({\mathrm{LDC}}_{\mathrm{Obs}}^i-{\mathrm{LDC}}_{\mathrm{Pre}}^i\right)}^2}{\sum_{i=1}^N{\left(\left|{\mathrm{LDC}}_{\mathrm{Pre}}^i-{\mathrm{LDC}}_{\mathrm{Obs}}^{\mathrm{mean}}\right|-\left|{\mathrm{LDC}}_{\mathrm{Obs}}^i-{\mathrm{LDC}}_{\mathrm{Obs}}^{\mathrm{mean}}\right|\right)}^2} $$

22 $$ \mathrm{MAE}=\frac{\sum_{i=1}^N\left|{\mathrm{LDC}}_{\mathrm{Pre}}^i-{\mathrm{LDC}}_{\mathrm{Obs}}^i\right|}{N} $$

23 $$ \mathrm{OI}=\frac{1}{2}\left(2-\frac{\mathrm{RMSE}}{{\mathrm{LDC}}_{\mathrm{Obs}}^{\max_{\mathrm{Obs}}^{\mathrm{min}}}-\frac{\sum_{i=1}^N{\left({\mathrm{LDC}}_{\mathrm{Obs}}^i-{\mathrm{LDC}}_{\mathrm{Pre}}^i\right)}^2}{\sum_{i=1}^N{\left({\mathrm{LDC}}_{\mathrm{Obs}}^i-{\mathrm{LDC}}_{\mathrm{Obs}}^{\mathrm{mean}}\right)}^2}}\right) $$

24 $$ \mathrm{OBJ}=\left(\frac{N_{{\mathrm{LDC}}_{\mathrm{tr}}}-{N}_{{\mathrm{LDC}}_{\mathrm{te}}}}{{\mathrm{No}}_{{\mathrm{LDC}}_{\mathrm{total}}}}\right)\frac{{\mathrm{MAE}}_{{\mathrm{LDC}}_{\mathrm{tr}}}}{{R^2}_{{\mathrm{LDC}}_{\mathrm{tr}}}}+\frac{2{N}_{{\mathrm{LDC}}_{\mathrm{te}}}\times {\mathrm{MAE}}_{{\mathrm{LDC}}_{\mathrm{te}}}}{{\mathrm{No}}_{{\mathrm{LDC}}_{\mathrm{total}}}\times {R^2}_{{\mathrm{LDC}}_{\mathrm{te}}}} $$

where LDC_Pre_ and LDC_Obs_ are the estimated and observed values of LDC, respectively; LDC^mean^ indicates the average estimated values; and *N* is the length of observation dataset (Gandomi et al. 2010; Ghaemi et al. 2019).

## LDC prediction result and discussion

### Comparison of different models

In this paper, the accuracy of a new equation that was obtained by CSA (EPR-CSA) is evaluated for the prediction of LDC. The inputs employed in the proposed model are velocity, shear velocity, channel width, and flow depth. The models were calibrated (i.e., trained) by using 103 datasets (about 70% of total dataset) while the remaining data (46 data) were utilized for validating the proposed model. The extracted equation is as follows:

25 $$ LDC=+1.45142\frac{\kern.2em {U}^{1.33648}\kern.2em }{B^{1.64538}{U_{\ast}}^{1.65004}}\kern.2em \exp \kern.2em \left(-1.34848H+1.42431U-1.41526{U}_{\ast}\right)+1.20883\frac{\kern.2em {U}^{1.77612}\kern.2em {H}^{0.91413}{B}^{0.67221}}{{U_{\ast}}^{0.61864}}\kern.2em \exp \kern.2em \left(-0.98949{U}_{\ast}\right)+1.21398\kern0.5em $$

In the present study, the results corresponding to the abovementioned benchmarks of 16 regression and AI-based equations were initially compared with the predictive equation obtained by EPR-CSA, and this can be difficult to have a comprehensive and comparable assessment. As asserted by Henseler et al. (2009) and Hair et al. (2013), the acceptance condition of models’ performance is determination coefficient (*R*^2^) ≥0.75, and it means that the response variable can be perfectly explained with insignificant error by the predictor variables. In this sense, eight equations provided by Seo and Cheong (1998), Deng et al. (2001), Li et al. (2013), Zeng and Huai (2014), Disley et al. (2014), Wang and Huai (2016), EPR (2018), and CSA are selected based on their determination coefficients calculated (higher than 0.75 (Table 4)).

**Table 4:** Satisfactory of utilized methods for LDC prediction

Equation	*R*^2^	Satisfy
Elder (1959)	0.42	No
Fischer (1967)	0.14	No
Liu (1977)	0.19	No
Koussis and Rodriguez-Mirasol (1988)	0.13	No
Iwasa and Aya (1991)	0.29	No
Seo and Cheong (1998)	0.76	Yes
Deng et al. (2001)	0.75	Yes
Kashefipour and Falconer (2002)	0.74	No
Sahay and Dutta (2009)	0.73	No
Etemad-Shahidi and Taghipour (2012)	0.55	No
Li et al. (2013)	0.75	Yes
Zeng and Huai (2014)	0.76	Yes
Disley et al. (2014)	0.77	Yes
Antonopoulos et al. (2015)	0.001	No
Wang and Huai (2016)	0.75	Yes
Alizadeh et al. (2017c)	0.68	No
Rezaie-Balf et al. (2018)	0.79	Yes
Kargar et al. (2020)	0.61	No
Memarzadeh et al. (2020)	0.69	No
Present study-CSA	0.80	Yes

To confirm the robustness of the proposed approach, EPR-CSA, this section presents the performances of the selected methods to estimate LDC. To evaluate the merits of the proposed method, a plethora of evaluation metrics, as expressed by Eqs. (18)–(24), is selected to illustrate the predictive performance criteria achieved in the calibration and validation stages. The predictive capability of the EPR-CSA and equations provided by previous research in LDC prediction is shown concisely in Tables 5 and 6.

**Table 5:** Evaluation of the proposed models at calibration stage

Method	*R*^2^	RMSE	WI	NSE	MAE	OI
Seo and Cheong (1998)	0.75	101.223	0.929	0.709	54.435	0.820
Deng et al. (2001)	0.74	95.600	0.914	0.740	46.436	0.8383
Li et al. (2013)	0.76	92.580	0.923	0.757	43.907	0.847
Zeng and Huai (2014)	0.71	113.601	0.846	0.634	46.923	0.778
Disley et al. (2014)	0.58	134.154	0.748	0.489	52.646	0.699
Wang and Huai (2016)	0.63	122.431	0.815	0.575	52.457	0.746
Rezaie-Balf et al. (2018)	0.80	88.710	0.945	0.776	47.076	0.858
Present study-CSA	0.78	88.756	0.936	0.776	39.538	0.858

**Table 6:** Evaluation of the proposed models at validation stage

Method	*R*^2^	RMSE	WI	NSE	MAE	OI
Seo and Cheong (1998)	0.76	95.534	0.922	0.659	60.258	0.772
Deng et al. (2001)	0.75	83.761	0.927	0.738	49.665	0.818
Li et al. (2013)	0.75	82.132	0.921	0.748	44.856	0.824
Zeng and Huai (2014)	0.76	89.425	0.884	0.701	48.869	0.797
Disley et al. (2014)	0.77	93.018	0.863	0.677	48.997	0.782
Wang and Huai (2016)	0.75	91.311	0.878	0.688	49.830	0.789
Rezaie-Balf et al. (2018)	0.79	81.548	0.940	0.751	48.529	0.827
Present study-CSA	0.80	77.557	0.935	0.775	42.987	0.841

Conventional benchmarks (*R*^2^, RMSE, NSE, WI, OI, as well as MAE) were applied for LDC prediction in the calibration stage, and the quantitative comparison of performances is shown in Table 5. Accordingly, by performing a comparison between the eight selected equations, the LDC prediction equation, which was observed by using the EPR model (proposed by Rezaie-Balf et al. 2018), had the highest level of accuracy with respect to statistical metrics (e.g., highest WI = 0.945 and *R*^2^ = 0.80, lowest RMSE = 88.71) in comparison with other predictive equations. After that, Eq. (25) achieved by EPR-CSA with minor difference from the EPR model in terms of RMSE (88.75), NSE (0.776), and *R*^2^ (0.787) ranked second.

In the case of validation dataset, it is apparent from Table 6 that Eq. (25) provided with EPR-CSA yielded the greatest precision (i.e., generally largest *R*^2^, OI, WI, and lowest RMSE) compared to the other approaches illustrating the crow search algorithm as a sturdy technique to enhance the EPR accuracy. For instance, as seen in Table 5, contrary to the results of the validation stage, the EPR-CSA model with lower MAE (11.41%) and higher OI (1.69%) in comparison with the EPR model with MAE = 48.52 and OI = 0.827 outperformed the EPR model. Moreover, Seo and Cheong’s (1998) equation with respect to NSE (0.659), MAE (60.25), and OI (0.772) could not estimate the LDC values compared to that of other methods, such as Wang and Huai (2016) (NSE = 0.688, MAE = 49.83, and OI = 0.789) and Disley et al. (2014) (NSE = 0.677, MAE = 48.99, and OI = 0.782)

**Fig. 4:**
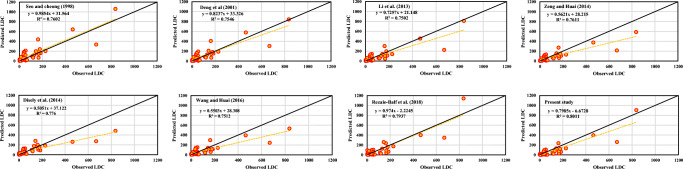
Scatterplot of LDC values of the predicted versus the observed

Furthermore, to gain more meticulous comprehension of the EPR-CSA model’s performance, the goodness-of-fit and Pearson’s correlation coefficients (*R*) of the observed LDC values versus the predicted ones are demonstrated by Fig. 4 for the validation dataset. Scatterplots confirm the best agreement between the output and predicted values. The determination coefficient (*R*^2^) with a linear fit equation *y* = *px* + *t* (*p* and *t* are taken into account as the gradient and the intercept on the *y*-axis, respectively) and a least squares regression (LSR) line have been presented in each sub-panel (Deo et al. 2016). As it is specified in Fig. 4, most of the LDC values predicted by the eight proposed equations were underestimated, and the estimated LDC values using EPR-CSA were closest to the perfect line and were in better agreement with corresponding observed values than others.

**Fig. 5:**
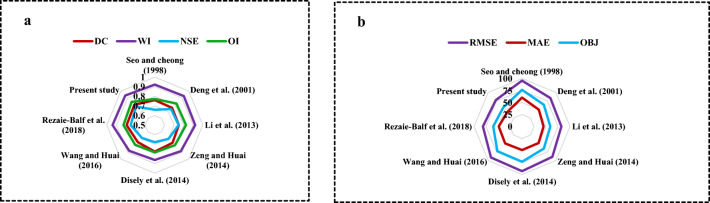
Polar plots related to the selected approaches **a** DC, WI, NSE, OI **b** RMSE, MAE, OBJ

Further analysis with the relative estimated error as presented on polar plots (Fig. 5) verifies the EPR-CSA model’s worthiness. As for the polar plots, the radial axis from origin illustrates the magnitude of the appraising benchmark calculated. Accordingly, it is obvious from Fig. 5a that the maximum values of evaluation metrics (*R*^2^, WI, NSE, and OI) generated by Eq. (25) were obtained by EPR-CSA. Moreover, the calculated values of RMSE, MAE, and OBJ for EPR-CSA were closer to the center of the regular octagon. These determined metrics, however, indicated the incapacitation of Seo and Cheong’s (1998) equation owing to the far distance from the regular octagon center in comparison with other alternative approaches (Fig. 5b).

**Fig. 6:**
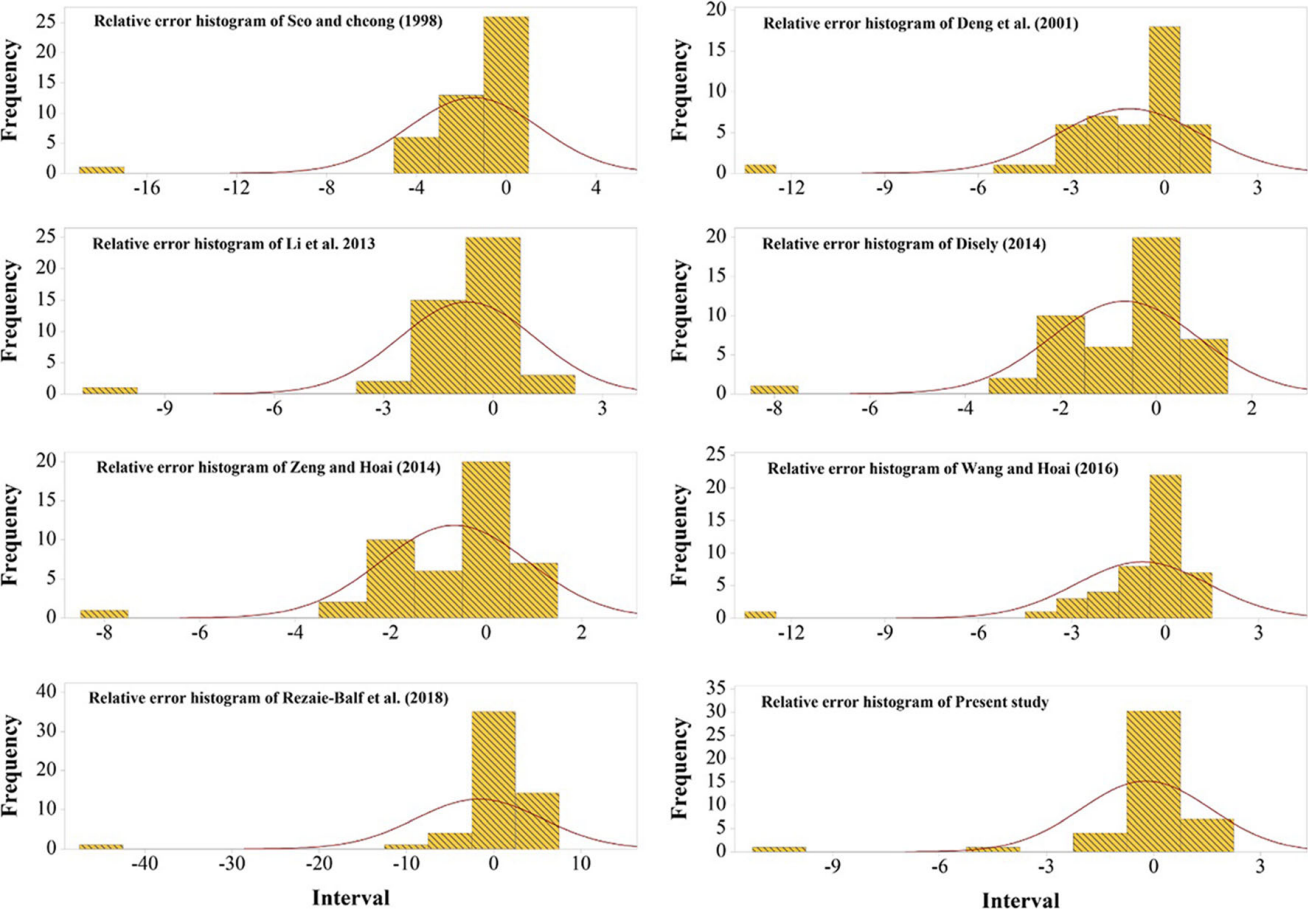
Relative forecasting error generated using proposed predictive equations for LDC prediction

To determine the error concentration in LDC estimation, the error histograms of the proposed approaches are plotted in Fig. 6. It can be seen that the error density for EPR-CSA aggregated around the zero roughly in an interval of −3 and 3, whereas this error density related to the EPR model is almost gathered around zero between −10 and 10. Consequently, the EPR-CSA performs more appropriately compared to the other equations.

### Partial derivative sensitivity analysis

The PDSA is considered one of the most prominent techniques to determine the pattern of changes in predictors by the superior approach (Azimi et al. 2017). It should be noted that the positive and negative values of PDSA denote the increasing and decreasing of the objective function, respectively. On the other hand, based on the PDSA, influence of decreasing or increasing of input variables on the output variable can be found. The positive PDSA value indicates the increasing trend of the LDC. In this technique, the relative derivative of the proposed equation is conducted for each input parameter (Rashki Ghaleh Nou et al. 2019).

**Fig. 7:**
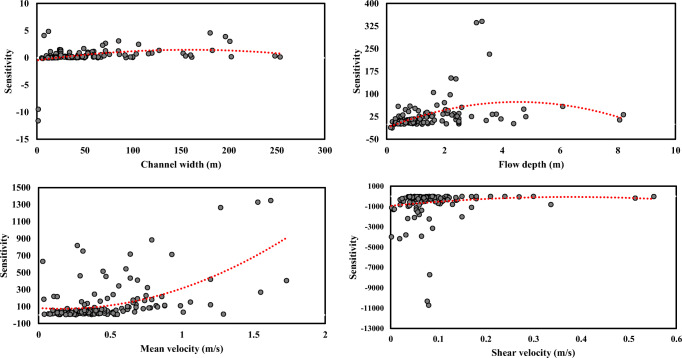
The results obtained by PDSA for LDC prediction using EPR-CSA

The results of PDSA for the input parameters (*B*, *H*, *U*, U_*_) for EPR-CSA, which could predict LDC values with a maximum level of accuracy, are shown in Fig. 7. In Fig. 7, all regression variables were plotted by means of a second-order polynomial. Accordingly, in the case of *U*, the calculated PDSA was positive, and by growing the *U* values, the sensitivity increased. Moreover, U_*_, *B*, and *H* versus sensitivity parameter’s behaviors were complicated and do not follow a particular trend.

### Reliability analysis

A major problem with the reliability of the predictive approach is calculating the multifold probability integral as a failure probability (*P*_f_) expressed as

26 $$ {P}_{\mathrm{f}}=\mathrm{Prob}\left[P(x)\le 0\right]={\int}_{p(x)\le 0}f(x)\mathrm{d}x $$

where *X* = [*x*_1_, *x*_2_, …, *x*_*n*_]^*T*^ and *T* and *X* are transposed and a vector of random variables, respectively, indicating the uncertainty of the structural quantities. The functions *P*(*X*) and *f*(*X*) represent the failure state and joint probability density function (PDF) of *X*, respectively. The negative values of *P*(ξ) (*P*(*x*) ≤ 0) reveal the integration domain which covers the failure set. As argued by Cardoso et al. (2008), the assessment of Eq. (26) is too difficult owing to some difficulties (Cardoso et al. 2008) including

1. Determining *P*(*X*),
2. Conducting the multidimensional integration of *P*(*X*) in the domain, and
3. Evaluating Eq. (26) either when the number of random variables rises or when the shape of failure regions is complicated (Cardoso et al. 2008).

These problems for calculating Eq. (26) can be essential factors to implement different approximation techniques. Generally, simulation is taken into account as a useful approach to perform experiments in a laboratory or on a digital computer to model the system behavior. Usually, simulation models output simulated data, which must be treated statistically for estimating the future treatment of that system. MCS is one of the appropriate tools that is usually applied for a number of problems, including random variables with proposed suitable probability distribution. By means of statistical sampling methods, random variables are generated based on the corresponding probability distribution. These values are treated similar to experimental datasets and are recruited to determine a sample solution. By repeating this process and generating various sample datasets, dozens of sample solutions can be obtained. Following this, the statistical analysis of the sample solutions is conducted. Thus, it can be said that the result of MCS approach depends on the length of the samples used.

In this study, the fundamental idea is that random values corresponding to the original variables, which are based on their appropriate probability distribution, are sampled, and the number of failure samples (*N*_f_) is determined. Afterward, the failure probability (*P*_f_) is calculated as follows (Mahadevan 1997; Cardoso et al. 2008):

27 $$ {P}_{\mathrm{f}}=\frac{N_{\mathrm{f}}}{N} $$

where *N* is the length of the samples and *N*_f_ indicates failure samples, and the failure probability is written as follows:

28 $$ {P}_{\mathrm{f}}=\frac{1}{N}{\sum}_{i=1}^NI\left(g(x)\right) $$

where *I*(.) denotes the failure area identifier, and the values of 0 and 1 show the safe and failure regions, respectively

if $$ g(x)\le 0 $$

29 $$ I\left(g(x)\right)=1 $$

else

$$ I\left(g(x)\right)=0 $$

**Fig. 8:**
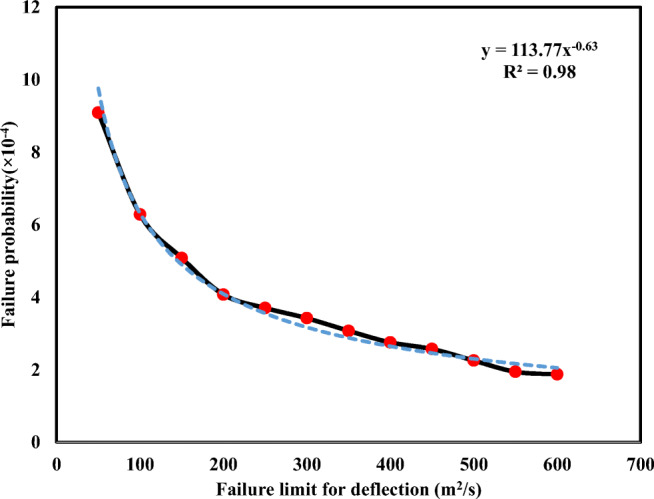
Failure probability in various failure states

In this section, the main aim is to determine the best distribution for the input variable. To gain this aim, since there are different probability distributions for the dataset with specific features, one of them defined in MATLAB was used to determine the proper distribution in the current study (Table 2). As mentioned earlier in Table 2, among the input variables, except the variable *B* that follows the lognormal distribution, the generalized extreme value distribution was selected as the best probability distribution for the remaining variables, namely *H*, *U*, and U_*_. Additionally, 1,000,000 samples for each of the input variables, based on their own distributions, were produced in order to estimate LDC values using Eq. (25). Eventually, the failure probability for a couple of failure-state values, including 50 to 600 m^2^/s, has been calculated, which is demonstrated in Fig. 8. Based on Fig. 8, by increasing the value of failure state, *P*_f_ decreased. It should be noted that a power-fitted function by *R*^2^ = 0.98 for the prediction of correct failure probability is more suitable than the other fitted functions, such as linear and exponential functions.

Furthermore, changes in average (*μ*) and standard deviation (*σ*) corresponding to each predictor variable’s appropriate distribution may have impact on the failure probability. On the other hand, this analysis evaluates each input variable’s influence on failure probability behavior of the proposed technique. Thus, in this research, the influence of changes in *B*, *H*, *U*, and U_*_ on the failure probability of LDC predictive equation is investigated. To achieve this target, values for each predictor’s average and standard deviation separately varied in the interval of their own 0.75 and 1.25 values. Moreover, three LDC values, including 50 m^2^/s, 100 m^2^/s, as well as 150 m^2^/s, were considered as failure states of failure probability.

#### Channel width effect

**Fig. 9:**
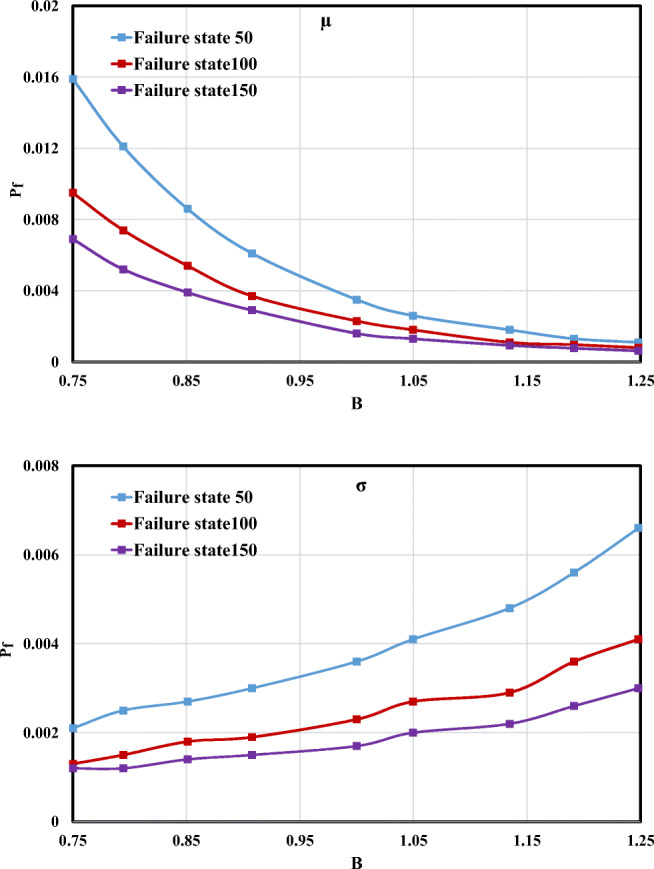
Failure probability changes for the different *μ* and *σ* values of *B*

Results of the failure probability changes versus the *μ* and *σ* of channel width (*B*) are presented in Fig. 9. Regarding Fig. 9, increasing the average of *B* leads to a decrease in failure probability. For instance, in the failure state 50 m^2^/s, the *P*_f_ value for *μ* = 2.64 m was equal to 0.016, and by increasing *μ* to 4 m, the calculated *P*_f_ had a decreasing trend by 0.002. In the case of standard deviation, *P*_f_ had an ascending trend when the *σ* values increased. In addition, the rising slope of the failure state 50 m^2^/s was roughly higher than that of the failure state 150 m^2^/s.

#### Flow depth effect

**Fig. 10:**
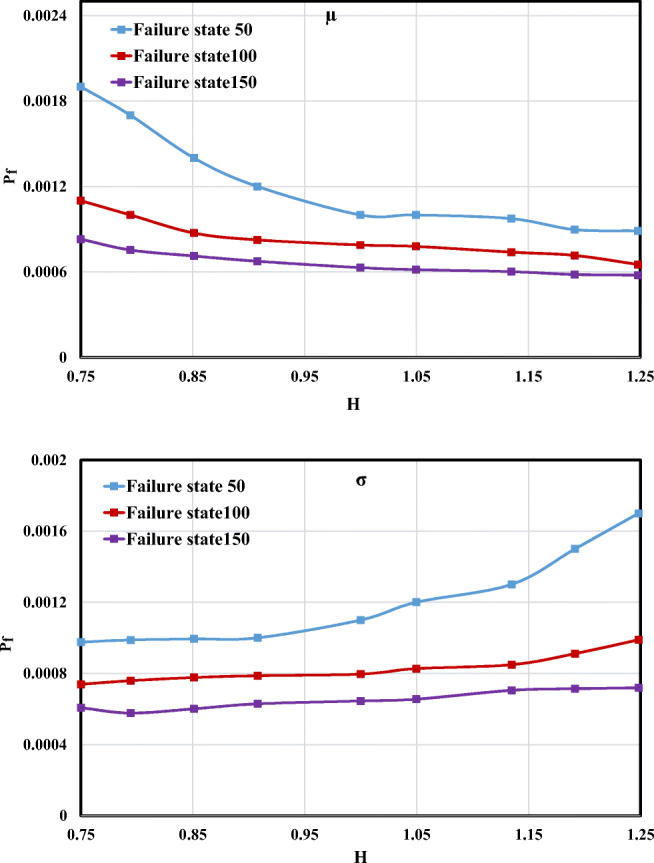
Failure probability changes for the different *μ* and *σ* values of *H*

Figure 10 illustrates the *P*_f_ changes versus the *μ* and *σ* of flow depth (*H*). As shown, *P*_f_ varies almost within the range of *μ* = 0.75 and *μ* = 1, which indicates that *μ* changing in this interval may have more impact on *P*_f_ in comparison to the range of *μ* = 1 and *μ* = 1.25. In contrast, the increasing value of *σ* caused failure probability to have a rising trend and obtained its highest value for all failure states such as having a *P*_f_ value of 0.0017 at a point with *H* = 1.25 for the failure state 50 m^2^/s.

#### Velocity effect

**Fig. 11:**
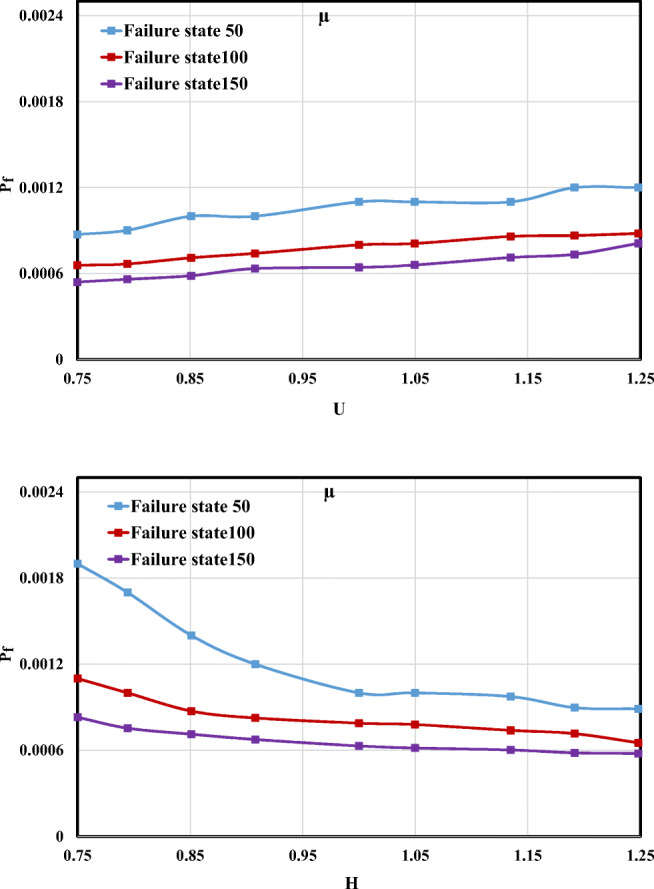
Failure probability changes for the different *μ* and *σ* values of *U*

The *P*_f_ changes versus the *μ* and *σ* of velocity (*U*) are shown in Fig. 11. Accordingly, in terms of *U*, it is obvious that increasing both values of *μ* and *σ* causes the failure probability to have a rising trend. However, the influence of increasing *σ* on the *P*_f_ was greater than *μ*. Additionally, for the failure state 100, the variation *P*_f_ in an interval of 75% and 125% of *μ* and *σ* was limited.

#### Shear velocity effect

**Fig. 12:**
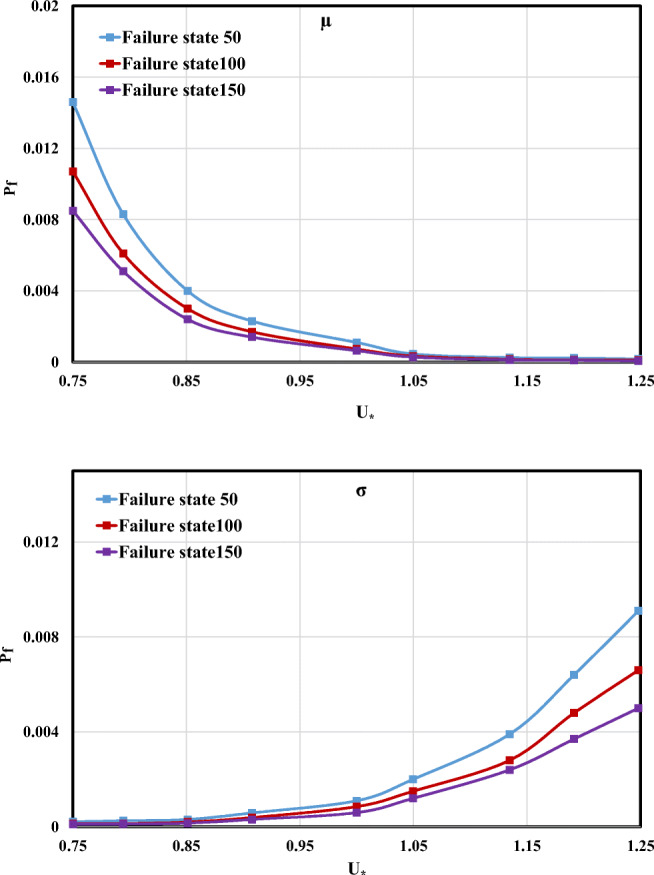
Failure probability changes for the different *μ* and *σ* values of U_*_

Similar to other input variables, the evaluation of *P*_f_ changes based on the *μ* and *σ* of shear velocity (U_*_) was performed. According to Fig. 12, an increase in the *μ* and *σ* values results in the descending and ascending trend of *P*_f_ value.

**Fig. 13:**
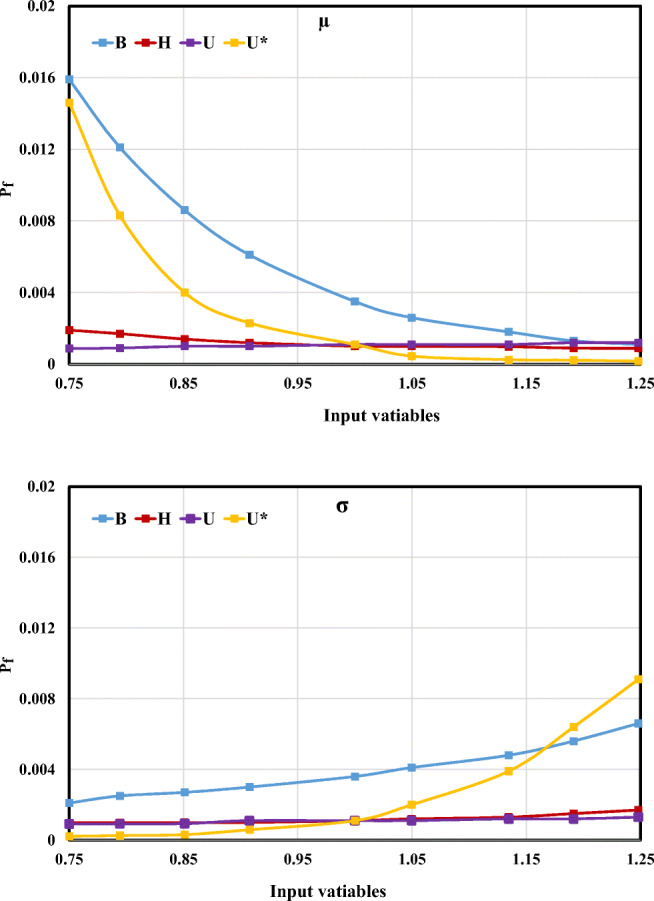
Failure probability variation for the different *μ* and *σ* input variables

It is clear that the highest values of failure probability in different *μ* and *σ* values of input variables belonged to the failure state 50 m^2^/s. Therefore, this failure state was selected to assess the maximum influence of input variables with respect to their average and standard deviation in this section. The *P*_f_ variation for different average values of input variables (*B*, *H*, *U*, U_*_) is shown in Fig. 13. From Fig. 13, it can be concluded that by increasing the average values of input variables, channel width (*B*) has the highest importance with respect to failure probability. In case of increasing *σ* between 75 and 115% of standard deviation for input variables, the *P*_f_ changes for *B* was more than those for U_*_. Additionally, the *σ* and *μ* variations of *H* and *U* had relatively the lowest *P*_f_ effect compared with *B* and U_*_.

## Conclusion

Accurate estimation of the LDC is one of the challenges in finding the distribution of pollution density. Due to this phenomenon’s nonlinearity and complexity, it is crucial to develop more accurate predictive approaches. To this end, this research implements and evaluates the efficiency of a kind of nature-inspired metaheuristic algorithm called crow search algorithm (CSA) to optimize the LDC equation coefficients provided using the EPR model. Outcomes of this comparison with respect to some evaluation metrics indicated that, among the existing equations, the proposed model EPR-CSA with a slight difference from the EPR model in terms of RMSE and WI had an acceptable accuracy in the calibration stage. In the case of validation dataset, recruiting the obtained equations by CSA illustrated that Eq. (25) could provide an acceptable estimation of LDC values for natural rivers with the lowest RMSE (77.57) and MAE (42.987). Eventually, comparing the results of LDC equations by applied evaluation benchmarks and diagnostic plots confirms the efficiency and robustness of the EPR-CSA versus other existing equations.

As a result, it can be concluded that CSA can be an alternative and promising estimation approach for complicated problems such as LDC prediction. Evaluating the pattern of input variables in LDC prediction reveals that the calculated value of PDSA related to *U* was positive, and increasing the value of *U* has an outstanding influence on growing the PDSA value. In addition, reliability analysis of the propose equation was performed by applying MCS. Determining the failure probability for several failure states containing 50 to 600 m^2^/s showed that, by increasing the value of the failure state, *P*_f_ is decreasing. Moreover, the influence of the input variables on the failure probability was assessed. According to the results, *σ* and *μ* changes for channel width (*B*) had the most effect on the *P*_f_ compared to those of other input variables.

## Data Availability

The datasets used during the current study are available from the corresponding author on reasonable request.

## Nomenclatures

AI: Artificial intelligence
ANFIS: Adaptive neuro-fuzzy inference system
ANN: Artificial neural network
ARE: Average relative error
*B*: Channel width
BA: Bee algorithm
BN: Bayesian network
CSA: Crow search algorithm
DE: Differential evolution
DR: Discrepancy ratio
EPR: Evolutionary polynomial regression
*H*: Flow depth
GA: Genetic algorithm
GC: Granular computing
GEP: Gene expression programming
GP: Genetic programming
GPR: Gaussian process regression
ICA: Imperialist competitive algorithm
LDC: Longitudinal dispersion coefficient
LMA: Levenberg–Marquardt algorithm
MAE: Mean absolute error
MARS: Multivariate adaptive regression splines
MCS: Monte Carlo simulation
ME: Mean of the absolute error
MER: Mean error rate
MLR: Multivariate linear regression
MT: Model tree
NE: Normalized error
NF-GMDH: Neuro-fuzzy–based group method of data handling
NSE: Nash–Sutcliffe efficiency
NLR: Nonlinear regression
*P*_f_: Failure probability
RAE: Relative absolute error
RMSE: Root mean square error
SE: Standard error
SVM: Support vector machine
SVR: Support vector regression
*U*: Velocity
*U*_∗_: Shear velocity
